# 1st ASCS: Expanding the ISCB Student Council Symposia to Asia

**DOI:** 10.12688/f1000research.135767.1

**Published:** 2023-06-20

**Authors:** Aayush Grover, Arsalan Riaz, Syed Muktadir Al Sium, Fatma B. Dincaslan, Sanjana Fatema Chowdhury, Gabriel J Olguin-Orellana, R. Gonzalo Parra, Pradeep Eranti

**Affiliations:** 1 Department of Computer Science, ETH Zurich, Zurich, Switzerland; 2 Swiss Institute of Bioinformatics (SIB), Zurich, Switzerland; 3 CECOS-RMI Precision Medicine Lab, Peshawar, Pakistan; 4 Industrial Microbiology Research Division, BCSIR Chattogram Laboratories, Bangladesh Council of Scientific and Industrial Research (BCSIR), Dhaka, Bangladesh; 5 Institute for Health Innovation and Technology, 117599, Singapore; 6 Department of Biomedical Engineering, National University of Singapore, 119077, Singapore; 7 Biological Research Division, BCSIR Dhaka Laboratories, Bangladesh Council of Scientific and Industrial Research (BCSIR), Bangladesh, Dhaka, Bangladesh; 8 Center for Bioinformatics, Simulation and Modeling (CBSM), Faculty of Engineering, Universidad de Talca, 1 Poniente 1141, Talca, Chile; 9 Laboratorio de Bioinformática y Química Computacional, Departamento de Medicina Traslacional, Facultad de Medicina, Universidad Católica del Maule, Talca, Chile; 10 Barcelona Supercomputing Center, Barcelona, Spain; 11 Universite Paris Cite, Inserm, UMRS-1124, Group of Genomic Epidemiology of Multifactorial Diseases, Paris, France

**Keywords:** ISCB Student Council, bioinformatics, computational biology, virtual, Asia

## Abstract

Since 2004, the ISCB Student Council (ISCB-SC) has successfully organized Student Council Symposia across several continents, including North America, Latin America, Europe, and Africa, as well as local events led by more than 25 Regional Student Groups (RSG) across the world. The ISCB-SC Symposia provide students and early career researchers the chance to showcase their work at an international venue in a format that includes keynote talks, round table discussions, workshops, and more. After several efforts spanning several years to build enough critical mass in the region, we have successfully organized the first Asian Student Council Symposium (1st ASCS). This article discusses the organizational details of this unprecedented event, the challenges faced, and the lessons learned.

## Introduction

The International Society of Computational Biology – Student Council (ISCB-SC) is a global organization that promotes bioinformatics and computational biology among students and early career researchers. The fields of bioinformatics and computational biology are at the intersection of multiple fields, such as biology, chemistry, mathematics, and informatics. This inherent diversity requires interactions between students and young researchers from all the aforementioned fields and backgrounds. The ISCB-SC initiated the Student Council Symposia (SCSs) to facilitate such interactions and has been organizing them since then. ^1^^–^^4^ This Symposium initiative has also evolved into continental symposia, ^5^^–^^8^ starting with the European Student Council Symposium in 2010 ^1^ and continuing with the Latin American ^9^ and the African ^10^ editions in 2014 and 2015, respectively. To complement these global and continental efforts, we aim to extend the ISCB-SC presence in the Asian region, which is long overdue.

**Figure 1.**
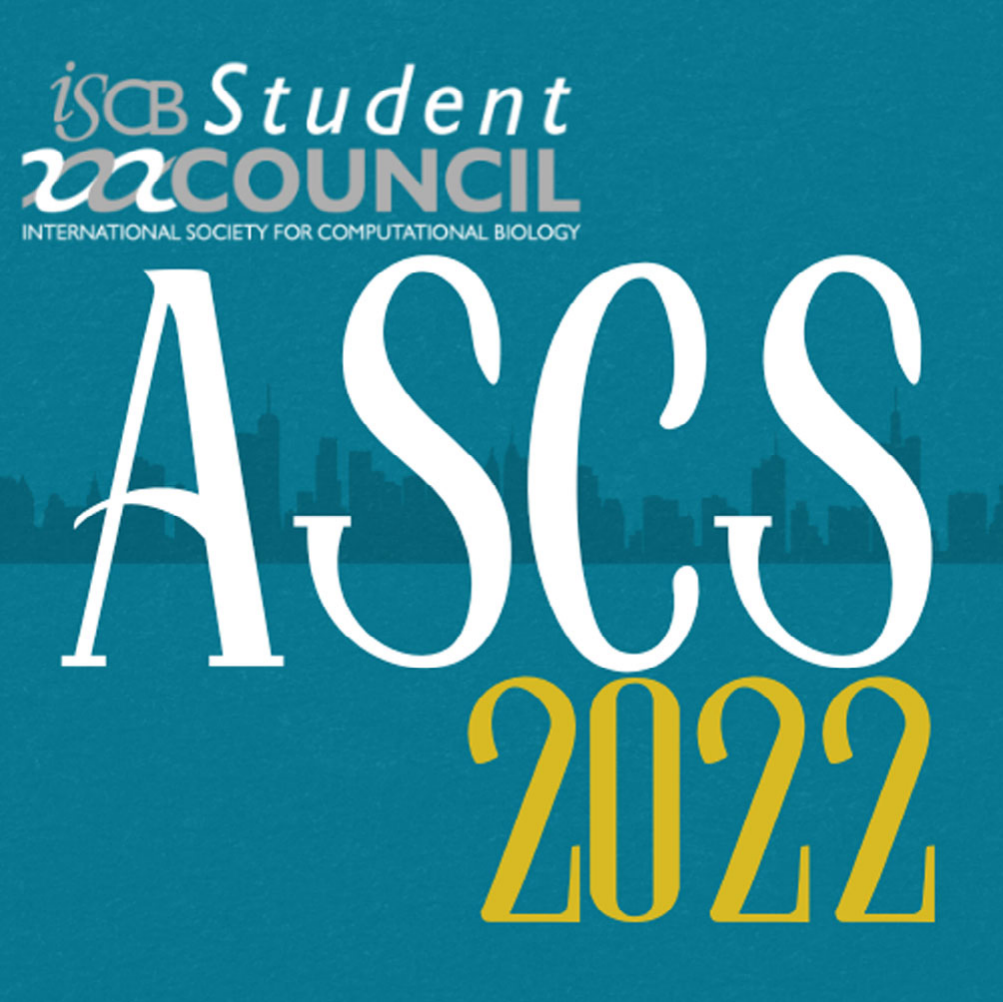
1st ASCS 2022 Logo.

Here, we reported the main highlights from the 1st ISCB Asian Student Council Symposium (ASCS; Figure 1) that preceded the in-person GIW XXXI/ISCB-Asia V conference (December 12-14, 2022, in Tainan, Taiwan). ASCS 2022 allowed students and young researchers in Asia to showcase their works on an international stage while also learning more about the field through keynotes, oral and poster presentations, workshops, and panel discussions. We are confident that the establishment of a periodic organization of ASCS will profoundly contribute to promoting the field of bioinformatics in the region, thereby leading to the development of the Asian ISCB-SC-related communities as it has happened with our other ISCB-SC continental Symposia.

## Format

The 1st Asian Student Council Symposium was organized as a two-day virtual event. The first day featured keynote talks, invited talks, student talks, and a round table discussion, while the second day featured workshops and a discussion session on the importance of preprints. This format allowed participants to get the most out of the practical workshops, where they could hone their skills in “Single-Cell Data Analysis” and “Reproducible Scientific Analysis.” The Poster Hall was open to participants with exclusive poster sessions and throughout the symposium, where participants could interact with the poster presenters and network. For the benefit of the participants, all relevant event information, including the schedule, abstracts, keynotes, and instructor profiles, was shared in the form of a program booklet. ^11^

Due to the COVID-19 pandemic, the ISCB and ISCB-SC organized their flagship activities virtually during 2020 and 2021, and they evolved into hybrid events in 2022. Implementing virtual and hybrid modalities has allowed ISCB to bring the content from its main conferences to the world and to promote research from regions where researchers cannot travel on-site due to economic and visa-related issues. Both modalities achieved an increase in equity, diversity, and inclusivity globally in computational biology. Due to these reasons, 1st ASCS was planned as a virtual event. The virtual setup also enabled participants to interact with one another and the speakers in the poster hall, where they could ask questions from the presenters, even anonymously, which helped to create a more inclusive environment and encouraged attendees to share their thoughts and ideas.

Based on the experience of the ISCB-SC with its other continental symposia, we deployed a robust outreach campaign to promote the 1st ASCS. We set up our symposium’s presence on four social media platforms, *i.e.*, Facebook, Instagram, LinkedIn, and Twitter, to communicate the symposium details, including announcing different aspects of the event, such as keynote speakers and key dates. This communication strategy was also supported by the ISCB Regional Student Groups (RSGs), the local nodes of the ISCB-SC, to promote computational biology in their respective regions. The Organizing Team of ASCS 2022 led the promotional campaign and amplified the campaign, with the help of local RSGs, ^12^ through multiple local communication channels for better reach.

**Figure 2.**
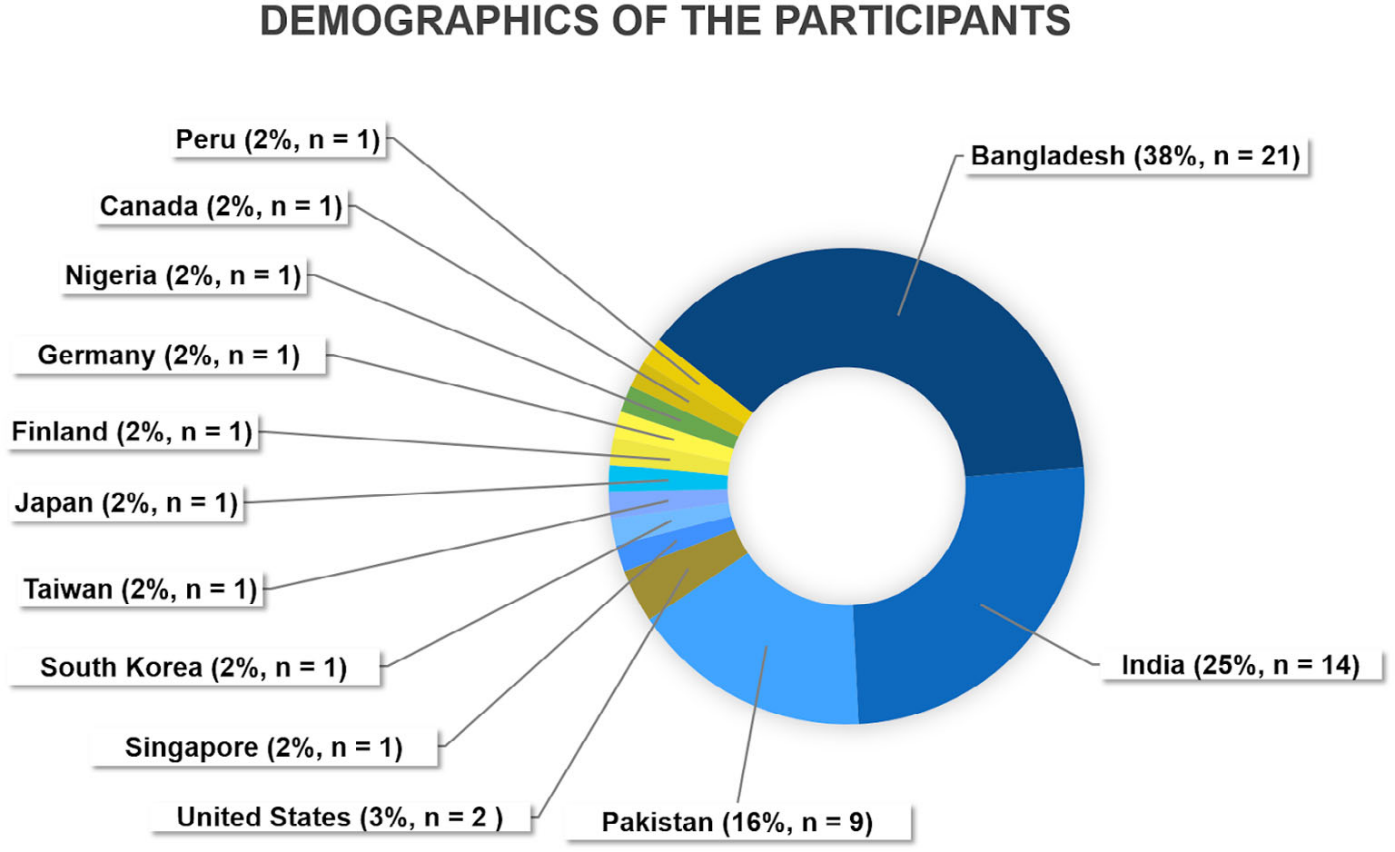
Demographics of the 1st ASCS participants.

Our main objective was to make ASCS 2022 as accessible as possible. The event was free to attend for ISCB members and those who had registered for the GIW XXXI/ISCB-Asia V conference, and it had a nominal fee for non-ISCB members. It is worth mentioning that this fee was tiered based on the country’s economic indicators, including Gross Domestic Product, Gross National Income, Wealth per capita, and Median Wealth per capita ( https://www.iscb.org/membership-dues). Such an approach resulted in 79 registrations, of which 80% of the participants, *i.e.* 63 of 79 registrations were from Bangladesh, India, and Pakistan ( Figure 2), where we have a large ISCB-SC presence. It is worth noting that there were several attendees from other geographical regions in the world showing that this event not only serves to expand the community in Asia but also to further connect it with the international scene.

## Keynote talks

The 1st ASCS hosted two keynote lectures on single-cell omics analysis and computational cancer genomics. Both topics were selected upon considering the recent trends and scientific interests in computational biology research. The opening keynote speaker Dr. Shyam Prabhakar from the Genome Institute of Singapore delivered the talk on “Spatial and Single Cell Omics: Algorithms and Applications” whereas the closing keynote speaker Dr. Sadaf Mughal from the DKFZ German Cancer Research Center spoke on “Comprehensive Molecular Characterisation of Soft Tissue Sarcoma”. Through these two keynotes, attendees not only familiarized themselves with the topics of single-cell and cancer genomics but also learned about the state-of-the-art research on these topics that is being performed at top research institutions.

### Spatial and single cell omics: algorithms and applications – Dr. Shyam Prabhakar

Dr. Prabhakar started his talk by introducing single-cell omics, how the field has been growing since 2009 ( *i.e.*, profiling one cell to, now, a million cells), why it is powerful, and why it is not the ultimate technology ( *i.e.*, how it evolves towards spatial omics to understand tissue dynamics and cell-to-cell communication for a given sample). He outlined how to filter some cells to improve input data by using the SampleQC tool they developed. For example, he compared two papers used for misclassification and misclaiming of novel subgroups in a research article ^13^ due to not eliminating cell debris at the early stages of analysis *versus* how stricter data quality parameters strengths reveal the heterogeneity of cells in their work. ^14^ After explaining how important it is to identify fresh cells from cell debris before downstream analysis, he switched to the challenges of spatial omics data analysis. Two major tasks were identified – cell type clustering and tissue domain segmentation. The discussion was based on the drawbacks of popular methods like the Hidden Markov Model (HMM) and Deep Neural Networks (DNN) used to resolve some challenges despite partial misassumptions. In summary, Dr. Prabhakar explained the importance of collaboration between disciplines, being careful in the selection of commonly used algorithms, choosing/developing simple methods where applicable to reduce variation and increase reproducibility, early quality control of sample/data for improving downstream analysis and accuracy of following interpretation of the results for single cell omics.

### Comprehensive molecular characterisation of soft tissue sarcoma – Dr. Sadaf Mughal

Adult Soft tissue sarcoma (STS) is a complex and heterogeneous group of malignancies that account for about 1% of all human cancers. Dr. Mughal started her talk by explaining the Leiomyosarcoma (LMS) subtype of STS and how her group identified significantly mutated genes in this subtype using Whole Exome Sequencing, RNA-Seq, and Copy Number Alterations. ^15^ Further, pathway analysis and studying somatic mutational signatures helped identify the effectiveness of treatments against LMS cell lines. Most STS treatments are based on cytotoxic drugs, except Pazopanib. Dr. Mughal then showed how pazopanib efficacy in STS can be predicted using gene expression. Further, 40 receptor tyrosine kinase (RTK) genes were selected, and their mutation status was assessed. The transcriptomic analysis showed a higher Pazopanib efficacy predictor to be more predictive in the presence of high expression of KDR and NTRK3 genes and low expression of the IGF1R gene. This would be essential to identify patients benefiting the most from pazopanib treatment. Finally, Dr. Mughal explained her analysis of the DNA methylation data from TCGA’s Sarcoma dataset to show that immune-cell infiltration differs across and within Sarcoma subtypes. ^16^

The key takeaway from this very informative keynote talk was that sarcoma subtypes have high heterogeneity, which calls for integrative analysis using genetic and epigenetic datasets to improve our knowledge of sarcoma genesis.

## Discussion sessions

The 1st ASCS also had two discussions: a round table discussion on women in Computational Biology on Day 1 and the importance of preprints on Day 2.

### Women in computational biology

The thought of not much noticeable presence of Asian women in computational biology made us arrange a round table discussion on “Asian Women in CompBio: Career Talk and Open Discussion.” We believe that it is important to openly discuss the issues faced by Asian women in this field and to brainstorm ways to inspire the future generation of scientists. To facilitate this, we hosted four successful researchers at the round table discussion: Dr. Farzana Rahman, a computer scientist and academic at Kingston University London, UK; Dr. Melike Dönertaş, a computational biologist, currently junior group leader, working on aging and age-related diseases at Leibniz Institute on Aging, Germany; Dr. Aishwarya Alex, a Software Project Lead at Roche Diagnostics Automation Solutions, Ludwigsburg, Germany; and Dr. Sayane Shome, a postdoctoral researcher in Machine Learning and Genomics at Stanford University, USA. Organising Team member Sanjana Fatema Chowdhury moderated the session.

The session started with a little introduction and experience sharing, their bioinformatics career journey. As former ISCB-SC leaders, all four speakers emphasized the value of volunteering at this kind of organization to gain experience and exposure to worldwide opportunities, including working in teams, networking with diverse groups of people, and understanding different cultures. This was followed by personal advice from the speakers towards women in STEM. Speakers suggested keeping all the options open and looking outside one’s boundaries by constantly connecting with people and forming new collaborations and networks. For example, Dr. Alex shared how she got into Science management in the private sector after completing her Ph.D.

One of the most common questions asked by the audience was how to find the right time and right opportunities within this field. The panelists recommended that one should attend large events and symposia, which are usually the best places to find people excited by similar research questions as yourself and who might be open to collaborating. Another option would be to browse through online platforms and use social media to contact the possibly right person in your field. One should not hesitate to reach out to people. As Wayne Gretzky rightly said – “ *You miss 100% of the shots you don’t take*”.

An anonymous attendee asked the question, which many of the students in the region have, whether getting into top-tier universities (based on rankings) matters a lot or not. The panel agreed that despite the possibility of good networking opportunities in some of these universities and making the hiring process easier in some instances, there are many opportunities in other good universities that cannot allocate a special budget to advertise and subsequently be ranked higher. The panel also emphasized the importance of applying to a lab having a healthy and balanced research environment instead of just applying due to the popularity of the school/institute.

A major concern that the moderator raised was that although we see more women in graduate schools (especially in Computational Biology), we see fewer leaders compared to the ratio of women involved in advanced research. We asked the panelists if there is a systematic bias against women leaders around the world and if they notice an improvement in the distorted leadership/higher-ranking positions. The panelists agreed that gender bias exists and encouraged more involvement of women in higher positions. On the other hand, they recommended not to be discouraged from applying to women supervisors despite generalized rumors against them. While the existence of bias was recognized, the panelists urged people not to blame systemic bias for everything. One needs to be more proactive and must promote their work. A key message here was the acknowledgment of the existence of such bias by the communities to raise awareness, take action and overcome together without assigning gender to abilities.

While it is of paramount importance to bridge the opportunity gap between men and women in STEM, many diverse strategies are already being taken in this direction, especially in computational biology, where we have Prof. Dr. Christine Orengo as the president of ISCB. Moreover, a large proportion of the ISCB Student Council leaders have been (Asian) women, as is the case of the panelists of this round table. Dr. Aishwarya Alex was the Chair of the ISCB-SC between 2019 and 2021, the same period in which Dr. Farzana Rahman and Dr. Sayane Shoeme were the ISCB Board of Directors Representative and the RSG Committee Chair, respectively whereas Dr. Melike Dönertaş was the Chair of the ISCB-SC in 2022.

### Importance of preprints

We invited Sciety ( https://sciety.org/) to discuss the importance of preprints and platforms like Sciety’s role in this. Mark Williams, Product Manager, and Shane Alsop, Community Manager and Outreach, presented the advantages of preprints, such as cost-free, open-access, rapid publicizing of research with a broader audience, career progression of early career researchers, citability, and ease to update. Moreover, they explained the “Publish-Review-Curate” model of eLife and where Sciety’s role between the preview and curation stages. The speakers also highlighted why it is essential to peer-review papers independently for research integrity and how this process helps to intercorporate writing and critical thinking ability of reviewer researchers to summarize the output scholarly. Following this, we discussed the current understanding of research and how social media platforms like Twitter (threads) might increase research visibility. Then a question was raised about how to choose the relevant ones considering the massive amount of preprints. This led to the appreciation of the curation process and recognition of the value of the diversity of peer reviewer/reviewer groups. In summary, we gained insight into the advantages and disadvantages of the preprint, the challenges and importance of the evaluation process, Sciety’s role in the preprint review and curation process, and how the research discovery, evaluation, and output process is evolving lately.

## ASCS: a platform for young researchers

**Figure 3.**
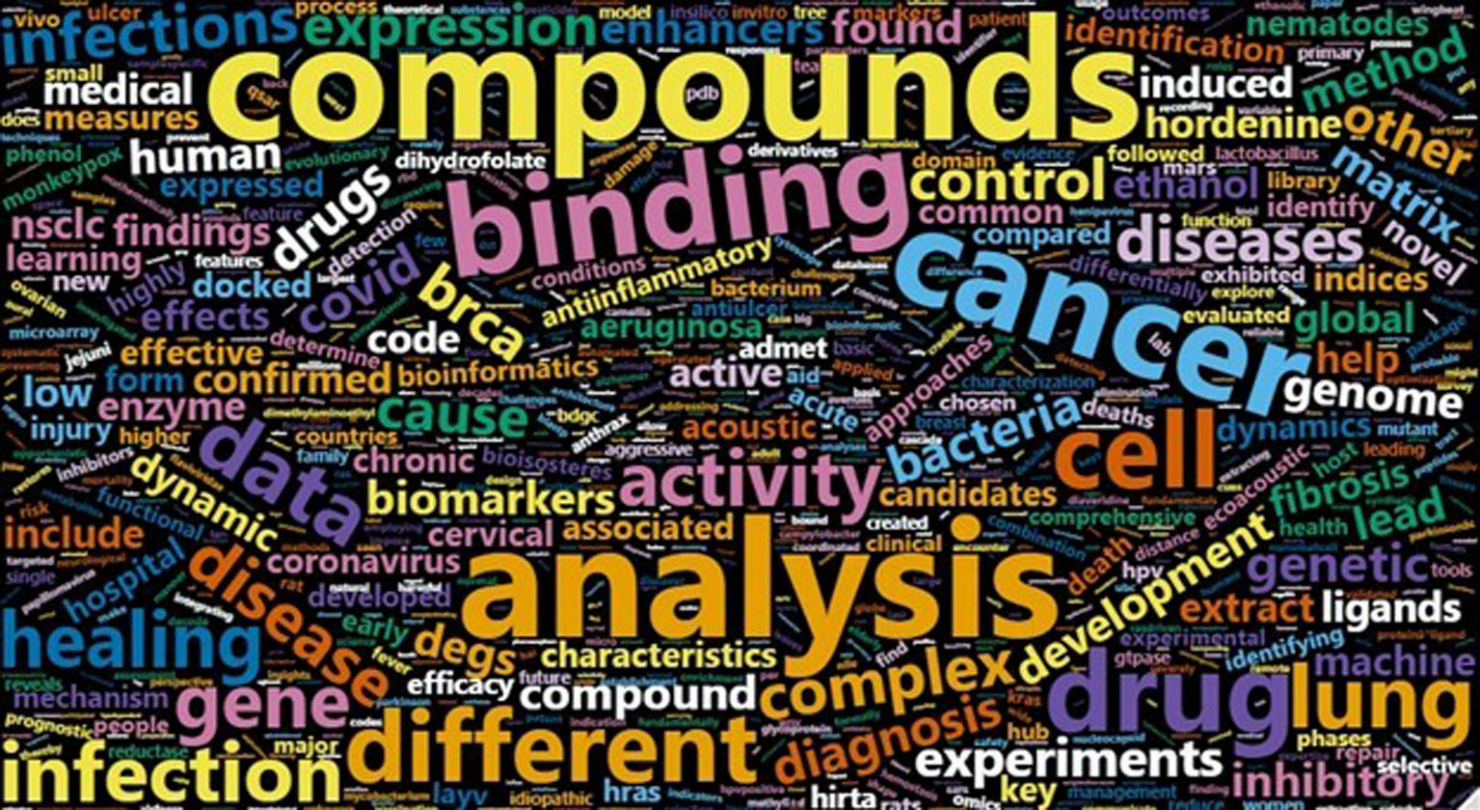
Word cloud of all submitted abstracts.

The 1st ASCS symposium provided a platform focused on Asia-based students and early-career researchers to present their cutting-edge research in computational biology. We received a total of 37 abstracts, out of which 25 abstracts were accepted as 6 oral talks, 10 flash talks, and 9 poster-only presentations. The selection of talks was based on a rigorous process that included calling for reviewers and reaching out to scientists and Ph.D. students with previous experience in reviewing. Interested reviewers were asked to review the background, objective, methodology, and findings sections of the abstracts for clarity, usability, uniqueness, relevance to the computational biology field, and completeness criteria. The word cloud in Figure 3 represents the diversity of research topics presented at the symposium. Most of the accepted abstracts were related to structural biology, more specifically molecular docking, with frequent occurrences of words such as compounds, analysis, and drugs. The symposium also featured a talk by Prof. Kenta Nakai of the University of Tokyo, Japan, who talked about the regulatory elements that are active globally in the human genome.

Recognitions for the best presentations across three categories were awarded: oral talks, flash talks, and posters. Nominations were made through Google form with access restricted to symposium participants, including organizers. Each response was allowed to vote for two oral presentations and two flash talks or poster presentations. Finally, the results were revealed in the closing ceremony on the second day of the symposium after discussing the importance of preprints. The best oral presentation prize went to Senuri De Silva from the National University of Singapore, Singapore, for the talk “A Strategized Machine Learning-based Approach for Extracting Robust Biomarkers from Proteomics Datasets: Identifying Novel Ovarian Cancer Markers”. This popular presentation received 80% of the total votes for the Oral presentation category. The best flash award went to Md. Asaduzzaman from the University of Rajshahi, Bangladesh, for the presentation on “A combined approach of Machine Learning Based QSAR, Molecular Docking, and Molecular Dynamics Simulation for Screening of Taiwanese Phytochemicals Against Cancer Target GTPase KRAS.” The best poster award was bagged by Rubaiat Ahmed from the University of Dhaka, Bangladesh, for the work on “Most frequently harbored missense variants of hACE2 across different populations exhibit varying patterns of binding interaction with spike glycoproteins of emerging SARS-CoV-2 of different lineages”. All three winners received a copy of the books including Illustrated Guide to Machine Learning, Ikigai, Think and Grow Rich, The Midnight Library, and The Wings of Fire.

## Workshops

The workshops aimed to provide attendees with hands-on experience on two topics: (1) single-cell sequencing data analysis and (2) reproducible scientific analysis. The organization team voted to decide on the workshop topics. Moreover, an online poll was conducted on social media platforms to get the participants’ views. The workshop topics were chosen to complement keynote lectures, making them easier to understand and implement.

### Single-cell data analysis

The first workshop focused on single-cell sequencing data analysis, which has gained more attention in recent years due to advancements in single-cell encapsulation, sequencing, and downstream analysis methods. Merve Kahraman from the Genome Institute of Singapore (GIS)/ASTAR introduced the basics of RNA sequencing and the advantages and disadvantages of single-cell experiments. She compared platforms and methods for various research questions and how to choose a particular method, depending on the sample and research question ( *i.e.*, sample compatibility, the requirement of deep characterization *versus* not). Moreover, she explained the concepts of doublets, drop-outs, ambient RNAs, barcodes, and UMIs. After delivering the introduction to both computational as well as the wet lab aspects of sequencing, Pratap Seshachalam from the National Cancer Center Singapore (NCCS) gave a hands-on tutorial on downstream analysis of Hepatocellular Carcinoma patient data ^17^ using Seurat-Guided Clustering Tutorial ( https://satijalab.org/seurat/articles/pbmc3k_tutorial.html). This included an introduction to Seurat, creating a Seurat object from a 10X Cell Ranger object, standard pre-processing ( *i.e.*, Quality Control metrics, selecting cells for downstream analysis), normalization of the data, identification of highly variable genes, data scaling, PCA, clustering, non-linear linear low dimensionality representation visualizations with UMA/tSNE, differential gene expression analysis, and cells assignments to clusters with known markers. He also demonstrated a demo for the downstream pathway analysis using EnrichR ^18^ for interested cluster features.

### Reproducible scientific analysis

Imagine that you made the most fantastic meal in your life once. Then, you will never be able to get even closer to that. Now, imagine this happening in research articles despite insisting on science as a discipline that requires consistency and replication of the research. The reproducibility issue is one of the most important discussions for science articles in recent years. It was shown, based on a report in 2016, 70% of researchers agreed that they failed to make each other’s experiment work. ^19^ Therefore, the second workshop focused on reproducibility, which is becoming increasingly important due to the crisis ( *or perhaps a revolution* ^20^) in science, and the hype about breakthroughs. While one must catch up with this fast-moving field of research, one should not do so by compromising on the quality of the research. The workshop was jointly offered, in The Carpentries style, by Dr. Batool Almarzouq from the University of Liverpool, Dr. Joel Nitta from the University of Tokyo, and Pradeep Eranti from Université Paris Cité. The workshop started with the importance of reproducibility, factors behind irreproducibility, explaining the conceptual difference between *“reproducible, replicable, robust, and generalizable,*” and introducing ideas to improve reproducibility. The workshop discussed the ideas and ways to improve reproducibility using RStudio and the tools available in its ecosystem. The tutorial continued teaching good project management practices ( *i.e.*, README, LICENSE, CITATION, file-naming, directory hierarchy, including packages and updates). This is followed by introducing how to author Quarto documents which have advanced features compared to RMarkdown documents using a live coding approach, then report generation using Quarto code chunks/documents, citations, and references, how to set up version control in RStudio by creating a git repository (also git workflow), and saving code-generated plots. Furthermore, they demonstrated how to collaborate *via* Github ( *i.e.*, authenticate and “push” the latest updates) as the final part of the workshop. The key takeaways from this workshop were consistent data generation and sharing for better research practices by taking advantage of free and open-source tools, being aware of reproducible principles right from the start of the project initiation, generating reproducible data analysis pipeline and integrating software analysis and textual reports for better reproducibility. Furthermore, the workshop material is freely available to participants and non-participants ( https://iscbsc.github.io/2022-12-11-ASCS-online/).

## Discussions

The 1st ASCS was a great success, thanks to the hard work of all the organizers and volunteers. Like any event and as experienced previously by the ISCB-SC leadership over almost 20 years, the success resulted from overcoming several challenges that led the organizing committee to learn valuable lessons and grow as a team. ^21^

### Challenges

The 1st ASCS event did not develop without challenges and setbacks. Firstly, Asia is the largest continent spread across 11 different time zones ranging from GMT+4 to GMT+10, thereby making it difficult to find a common time zone. We solved this issue by selecting the almost central time zone, *i.e.*, GMT+6. This timezone was also chosen for the ease of organizers since most were based in Bangladesh, India, and Pakistan. Another challenge was the diversity of the organizing team itself. While most organizing members resided in Bangladesh, India, or Pakistan, some stayed in Singapore, Europe, and the US. To ensure everyone could attend the weekly internal meetings, we met around 7 pm or 8 pm GMT+6 on the weekend, which worked for almost everyone. We documented the minutes of each meeting so that those who could not attend could also stay updated. Offline discussions also happened through Slack. Very unexpected challenges appeared during the ASCS project; for example, during the time zone planning, we realized that Doodle.com was not accessible in Bangladesh. We faced similar issues throughout the organization and a few novel obstacles and challenges that other ISCB-SC symposia did not encounter.

It was also essential for us to come up with designs and colors that represent the identities of all 48 Asian countries. By additionally considering colorblindness, we chose a palette consisting of #AEAEAE, #D8BA25, #0593B2, and #076478. We used the skyline in the logo to highlight the major landmarks in Asia, as shown in Figure 1. The logo was designed using Canva.

Lastly, since the registration payments were to be made using the ISCB platform, several participants from Bangladesh struggled with this due to the limited availability of international payment options in Bangladesh. Therefore, either the participants reached out to their friends and family at home and abroad that allow international transactions or to ISCB. To resolve this, ISCB handled payments for these participants separately. Organizing the 1st ASCS symposium made us aware of the difficulties that an event not based in Europe or North America can face. These lessons must be noted as they represent an inevitable burden on organizing teams that are not based on those regions.

### Lessons learned

Despite the challenges mentioned above, the 1st ASCS was a success. As this was the first time a continental ISCB-SC event was organized in Asia, several lessons were learned. Collaboration between researchers from different countries can be challenging due to language barriers, cultural differences, and political tensions, limiting the opportunities for sharing knowledge and resourcesacross borders. This was one of the main reasons why the organization of the 1st ASCS took such a long time compared to similar events in other continents. Most importantly, with the 1st ASCS, we demonstrated that science could unite nations that have experienced political tensions among them. Such events should be encouraged to promote collaborations between countries which can not only lead to exposure to different cultures but also develop strong connections outside the manmade boundaries. Therefore, we hope that events like the 1st ASCS can enable computational biology researchers from different countries to work together in the name of science. Apart from the organizers, we were quite happy to see participants from diverse nations leading to exciting discussions among international peers ( Figure 2).

With ASCS 2022, we realized that the need to promote computational biology in the Asian region is more pressing than we had thought. Participants showed an interest in only a few subfields of research, for example, structural bioinformatics. We observed nearly 50% (7 out of 16) of the student talks were on this topic due to the high expertise in structural bioinformatics in the Asian region, as opposed to the dominance of multi-omics, both bulk and single cell, in the European countries. One reason for this could be relatively cheaper experimental tools for structural bioinformatics in the Asian region than those needed for multi-omics studies. However, other fields need to be promoted widely so that young researchers are not biased toward a particular subfield of research. One way to enable this is the organization of events like ASCS 2022 that can help to promote under-represented subfields within the region. At the end of the event, we also conducted a short survey before handing out the participation certificates. This allowed us to reflect on the 1st edition of ASCS and identify its shortcomings. 56 participants filled out the survey, and overall the feedback was highly positive and encouraging (~98% of participants rated the event 4+ out of 5), with many suggesting that we should organize ASCS regularly. The key upgrade the participants suggested was improving the virtual platform, including live streaming on select Over-The-Top (OTT) platforms, increasing interactivity, providing more content, fixing technical issues, making all recordings open access, and increasing interaction between speakers and attendees. While we can improve the virtual platform and introduce live streaming, making recordings and live streaming depends on the speakers and their privacy standards. In order to make the event more interactive, one could offer a hybrid symposium which can also be explored.

## Conclusion

For almost 20 years, The ISCB-SC has been the largest community to gather together and nurture the next generation of computational biologists and bioinformaticians worldwide. ^12^ However, while some regions like Europe and the Americas have flourished and expanded considerably during this period, others are still in very early stages or have yet to develop. One of the most effective ways in which the ISCB-SC has found to promote the continental development of its community has been through the establishment of continental Symposia with a bi-annual frequency that strengthens the collaboration among existing RSGs in the region as well as to stimulate the creation of new ones. After its creation in 2004, the ISCB-SC celebrated its 1st Student Council Symposium (SCS) in 2005, ^22^ followed by the 1st European SCS in 2010, the 1st Latin American SCS in 2014, and the 1st African SCS in 2015. All these regions experienced significant expansion after these events were celebrated. The 1st ASCS in 2022 was the first ISCB-SC event that successfully promoted computational biology and bioinformatics in the broad Asian region, which will be remembered as a fundamental milestone in the ISCB-SC history. Despite the challenges faced by the organizing team that is natural for any group of people who start to organize such kind of events for the first time, ^21^ such as finding a common time zone, diversity of the team, and choice of the online platform, the team was able to overcome them and deliver a great event. The two-day event comprised keynote speakers, student talks, workshops, and panel discussions of great relevance. Although there is room for improvement in aspects like the virtual platform and social interactivity, the feedback received from the participants is very positive and encouraging. While this event was conducted online, we aim to move to a hybrid setting and hope to overcome the new challenges we might face successfully. All the details of our event can be found at https://www.ascs2022.iscbsc.org/. As the fields of computational biology and bioinformatics continue to develop in the Asian region, events like the 1st ASCS, focused on the youngest layer of our research system, will be of great value to enhance such community growth. We are confident that new RSGs will be created due to the successful organization of 1st ASCS and the establishment of cross-country collaborations among different RSGs and other scientific communities. The seed has been planted, and a new group of energetic, motivated, and bright Asian scientists has taken the lead to elevate the fields of computational biology and bioinformatics in Asia. Join us on this fascinating journey, and *Let science break barriers and connect different Asian countries among themselves and with the rest of the great ISCB-SC ecosystem*!

## Data Availability

No data is associated with this article.

## References

[1] Hassan M Namasivayam AA DeBlasio D : Reflections on a journey: a retrospective of the ISCB Student Council symposium series. *BMC Bioinformatics.* 2018;19:347. 10.1186/s12859-018-2369-x 30301451 PMC6176502

[2] Parisi D Olguín-Orellana GJ Draizen EJ : Nurturing tomorrow’s leaders: The ISCB Student Council Symposia in 2018. *F1000Res.* 2019;8: ISCB Comm J-34. 10.12688/f1000research.17739.1 30647915 PMC6329205

[3] Cuypers WL Dönertaş HM Grewal JK : Highlights from the 16th International Society for Computational Biology Student Council Symposium 2020. *F1000Res.* 2021;10: ISCB Comm J-443. 10.12688/f1000research.53408.1 34136128 PMC8182693

[4] Osorio-Mogollon C Grentzinger V Olguin-Orellana GJ : ISCB Student Council Symposium 2021, a virtual global venue: challenges and lessons learned. *F1000Res.* 2023;12:50. 10.12688/f1000research.129945.1 36704314 PMC9837453

[5] Monzon AM Hasenahuer MA Mancini E : Second ISCB Latin American Student Council Symposium (LA-SCS) 2016. *F1000Res.* 2017;6: ISCB Comm J-1491. 10.12688/f1000research.12321.1 28928937 PMC5580427

[6] Olguín-Orellana GJ Papadimitriou S Langtry Yáñez A : 6th European Student Council Symposium (ESCS): overcoming obstacles to enhance virtuality, connectivity, inclusivity and community engagement. *F1000Res.* 2021;10: ISCB Comm J-41. 10.12688/f1000research.40666.1 33537121 PMC7836082

[7] Akurugu WA Doughan A Adamu Bukari A-R : Highlights of the 3rd ISCB Africa Student Council Symposium 2019 in Ghana. *F1000Res.* 2020;9: ISCB Comm J-448. 10.12688/f1000research.24101.1 32518632 PMC7255899

[8] Castillo-Vilcahuaman C Valdivia C Osorio-Mogollón C : 4th ISCB Latin American Student Council Symposium: a virtual and inclusive experience during COVID-19 times. *F1000Res.* 2020;9: ISCB Comm J-1460. 10.12688/f1000research.28330.1 33363714 PMC7739094

[9] Parra RG Simonetti FL Hasenahuer MA : Highlights from the 1st ISCB Latin American Student Council Symposium 2014. Introduction. *BMC Bioinformatics.* 2014;16 Suppl 8(Suppl 8):A1. 10.1186/1471-2105-16-s8-a1 25955751 PMC4423572

[10] Souilmi Y Allali I Badad O : Highlights of the first ISCB Student Council Symposium in Africa 2015. *F1000Res.* 2015;4: ISCB Comm J-569. 10.12688/f1000research.6877.1 26998231 PMC4786895

[11] Riaz A Grover A Eranti P : Program Booklet of the 1st Asian Student Council Symposium. 2022 [cited 15 May 2023]. 10.5281/ZENODO.7934696

[12] Shome S Parra RG Fatima N : Global network of computational biology communities: ISCB’s Regional Student Groups breaking barriers. *F1000Res.* 2019;8: ISCB Comm J-1574. 10.12688/f1000research.20408.1 31508204 PMC6720036

[13] Wilk AJ Rustagi A Zhao NQ : A single-cell atlas of the peripheral immune response in patients with severe COVID-19. *Nat Med.* 2020;26:1070–1076. 10.1038/s41591-020-0944-y 32514174 PMC7382903

[14] Joanito I Wirapati P Zhao N : Single-cell and bulk transcriptome sequencing identifies two epithelial tumor cell states and refines the consensus molecular classification of colorectal cancer. *Nat Genet.* 2022;54:963–975. 10.1038/s41588-022-01100-4 35773407 PMC9279158

[15] Chudasama P Mughal SS Sanders MA : Integrative genomic and transcriptomic analysis of leiomyosarcoma. *Nat Commun.* 2018;9:144. 10.1038/s41467-017-02602-0 29321523 PMC5762758

[16] Simon M Mughal SS Horak P : Deconvolution of sarcoma methylomes reveals varying degrees of immune cell infiltrates with association to genomic aberrations. *J Transl Med.* 2021;19:204. 10.1186/s12967-021-02858-7 33980253 PMC8117561

[17] Sharma A Seow JJW Dutertre C-A : Onco-fetal Reprogramming of Endothelial Cells Drives Immunosuppressive Macrophages in Hepatocellular Carcinoma. *Cell.* 2020;183:377–394. e21. 10.1016/j.cell.2020.08.040 32976798

[18] Chen EY Tan CM Kou Y : Enrichr: interactive and collaborative HTML5 gene list enrichment analysis tool. *BMC Bioinformatics.* 2013;14:128. 10.1186/1471-2105-14-128 23586463 PMC3637064

[19] Baker M : 1,500 scientists lift the lid on reproducibility. *Nature.* 2016;533:452–454. 10.1038/533452a 27225100

[20] Vazire S : Implications of the Credibility Revolution for Productivity, Creativity, and Progress. *Perspect Psychol Sci.* 2018;13:411–417. 10.1177/1745691617751884 29961410

[21] Mishra T Parra RG Abeel T : The Upside of Failure: How Regional Student Groups Learn from Their Mistakes. *PLoS Comput Biol.* 2014;10:e1003768. 10.1371/journal.pcbi.1003768 25101799 PMC4125077

[22] Corpas M : Scientists & societies. *Nature.* 2005;436:1204–1204. 10.1038/nj7054-1204b 16144051

